# Effects of Lipids and Lipoproteins on Mesenchymal Stem Cells Used in Cardiac Tissue Regeneration

**DOI:** 10.3390/ijms21134770

**Published:** 2020-07-05

**Authors:** Yi-Hsiung Lin, Lin Kang, Wen-Han Feng, Tsung-Lin Cheng, Wei-Chung Tsai, Hsuan-Ti Huang, Hsiang-Chun Lee, Chung-Hwan Chen

**Affiliations:** 1 Division of Cardiology, Department of Internal Medicine, Kaohsiung Medical University Hospital, Kaohsiung Medical University, Kaohsiung 807, Taiwan; caminolin@gmail.com (Y.-H.L.); hans0426@gmail.com (W.-H.F.); k920265@gap.kmu.edu.tw (W.-C.T.); 2 Lipid Science and Aging Research Center, Kaohsiung Medical University, Kaohsiung 807, Taiwan; 3 Center for Lipid Biosciences, Kaohsiung Medical University Hospital, Kaohsiung 807, Taiwan; 4 Department of Biotechnology, Kaohsiung Medical University, Kaohsiung 807, Taiwan; 5 Department of Obstetrics and Gynecology, National Cheng Kung University Hospital, College of Medicine, National Cheng Kung University, Tainan 704, Taiwan; kanglin@mail.ncku.edu.tw; 6 Department of Internal Medicine, Kaohsiung Municipal Ta-Tung Hospital, Kaohsiung Medical University Hospital, Kaohsiung Medical University, Kaohsiung 801, Taiwan; 7 Orthopaedic Research Center, Kaohsiung Medical University, Kaohsiung 807, Taiwan; junglecc@gmail.com (T.-L.C.); hthuang@kmu.edu.tw (H.-T.H.); 8 Regeneration Medicine and Cell Therapy Research Center, Kaohsiung Medical University, Kaohsiung 807, Taiwan; 9 Department of Physiology, College of Medicine, Kaohsiung Medical University, Kaohsiung 807, Taiwan; 10 Faculty of Medicine, College of Medicine, Kaohsiung Medical University, Kaohsiung 807, Taiwan; 11 Department of Orthopedics, Kaohsiung Medical University Hospital, Kaohsiung Medical University, Kaohsiung 807, Taiwan; 12 Departments of Orthopedics, College of Medicine, Kaohsiung Medical University, Kaohsiung 807, Taiwan; 13 Department of Orthopedics, Kaohsiung Municipal Ta-Tung Hospital, Kaohsiung 807, Taiwan; 14 Department of Internal Medicine, School of Medicine, College of Medicine, Kaohsiung Medical University, Kaohsiung 807, Taiwan; 15 Institute of Medical Science and Technology, National Sun Yat-sen University, Kaohsiung 804, Taiwan

**Keywords:** cardiac tissue regeneration, lipid, lipid-lowering drug, lipoprotein, mesenchymal stem cell, Simvastatin

## Abstract

Mesenchymal stem cells (MSCs) have two characteristics of interest for this paper: the ability to self-renew, and the potential for multiple-lineage differentiation into various cells. MSCs have been used in cardiac tissue regeneration for over a decade. Adult cardiac tissue regeneration ability is quite low; it cannot repair itself after injury, as the heart cells are replaced by fibroblasts and lose function. It is therefore important to search for a feasible way to repair and restore heart function through stem cell therapy. Stem cells can differentiate and provide a source of progenitor cells for cardiomyocytes, endothelial cells, and supporting cells. Studies have shown that the concentrations of blood lipids and lipoproteins affect cardiovascular diseases, such as atherosclerosis, hypertension, and obesity. Furthermore, the MSC lipid profiles, such as the triglyceride and cholesterol content, have been revealed by lipidomics, as well as their correlation with MSC differentiation. Abnormal blood lipids can cause serious damage to internal organs, especially heart tissue. In the past decade, the accumulated literature has indicated that lipids/lipoproteins affect stem cell behavior and biological functions, including their multiple lineage capability, and in turn affect the outcome of regenerative medicine. This review will focus on the effect of lipids/lipoproteins on MSC cardiac regenerative medicine, as well as the effect of lipid-lowering drugs in promoting cardiomyogenesis-associated MSC differentiation.

## 1. Lipid-Induced Cardiac Tissue Damage

Increasing evidence is emerging to support that idea that lipids/lipoproteins cause lipotoxicity toward cardiac tissue, including cardiac muscle, valves, and blood vessels. Low-density lipoproteins (LDLs) have been reported to have a cytotoxic role in inducing atherosclerotic disease [1], as well as playing a role in chronic inflammatory disorder [2]. The most typical oxidized LDL (oxLDL) is known to have a considerable relationship with coronary artery disease (CAD) [3,4]. LDLs with negative charges can be separated into five subclasses, from L1 to L5, all exhibiting the ability of vascular cells to promote atherogenesis. Among them, L5, which carries the strongest negative charge among the LDLs of patient plasma, was found to be associated with an increased risk of cardiovascular disease, comparable to the risk of smoking [5], hypercholesterolemia [6], type 2 diabetes mellitus [7], and metabolic syndrome [8]. L5 contains higher total protein and triglycerides, but relatively few cholesterol esters, compared with L1, suggesting that the lipotoxicity of L5 activates the related various adhesion molecules and chemokines through cellular processes. Lectin-like oxidized LDL receptor-1 (LOX-1) has been shown to have a high binding affinity for negatively charged ligands, which facilitates downstream signaling transduction of, for example, the Bcl-2 family, leading to cell death [9]. The association of LDL and L5-LDL with the risk of coronary artery disease (CAD) is still being investigated, along with the mechanisms of how those lipoproteins affect related tissues and cells.

We have also reported that very low-density lipoprotein (VLDL) can be separated into five different types, defined by the negative charge and known as V1 to V5, with different biological functions. We found that V5 exhibited the greatest degree of cytotoxicity of all the VLDLs in the patient’s plasma, and that patients with metabolic syndrome had a higher percentage of VLDLs in the plasma than normal people [10]. Metabolic syndrome (MetS) patients had significantly higher percentages and higher concentrations of V5/VLDL than the normal population. An abnormally high content of V5-rich VLDL may increase the risk of diabetes, and related vascular lesions and cardiomyopathy. Dyslipidemia is easily link to atrial fibrillation (AF) [11,12], which can be the originated from several cardiovascular diseases, including heart failure, hypertension, myocardial infarction [11], valvular heart disease [13], and rheumatic heart disease [14]. We have shown that the VLDLs in MetS patients (msVLDL) can cause significant left atrial dilation compared with normal VLDLs in treated mice, along with a decreased ejection fraction, and accompanied by unprovoked AF in elderly msVLDL mice. Our evidence indicates the pivotal cytotoxic role of VLDL in cardiomyocyte and AF pathogenesis [12]. These data indicate the potential toxic nature of VLDLs and LDLs toward human tissues, especially cardiac tissue, which can be associated with the occurrence of cardiac disease.

Dyslipidemia is one of the main risk factors for coronary heart disease, and may in turn contribute to cardiomyopathy, and cardiac death. Dyslipidemia refers to abnormal levels of circulatory lipids (such as triglycerides, cholesterol, and/or fatty phospholipids) in the blood [15,16]. In developed countries, the majority of dyslipidemia is hyperlipidemia; that is, abnormally elevated lipids in the blood [17]. Dyslipidemia is a series of metabolic abnormalities, which are usually characterized by one or more of the following: elevated low-density lipoprotein (LDL-c) (>130 md/dL), elevated total cholesterol (>200 mg/dL), or elevated TG (>150 mg/dL) or low density lipoprotein (HDL-c) (male <40 mg/dL, female <50 mg/dL). When the values of one or both of the triglycerides and total cholesterol in the blood exceed the normal values, it is called hyperlipidemia. Hyperlipidemia is also closely related to many diseases, such as stroke, hypertension, diabetes, kidney disease, and other chronic diseases [18,19]. As the cholesterol (especially bad cholesterol) or triglyceride concentration increases, it can cause the abnormal function of vascular endothelial cells. 

The associations of dyslipidemia and other atherosclerotic diseases, i.e., cerebrovascular disease and peripheral arterial disease have also been well-investigated. There is evidence proving the relationship between the abnormal distribution of lipids and the risk of coronary artery disease (CAD), and it is an independent risk factor for cardiovascular diseases (CVD) [20]. In addition, hypertriglyceridemia and mixed dyslipidemia are associated with the accumulation of metabolic risk factors, such as hypertension (HTN) and obesity. These abnormally expressed blood lipids cause lipotoxicity to tissue and cell functions. 

## 2. Mesenchymal Stem Cells (MSCs) in Cardiac Tissue Regeneration

**Figure 1:**
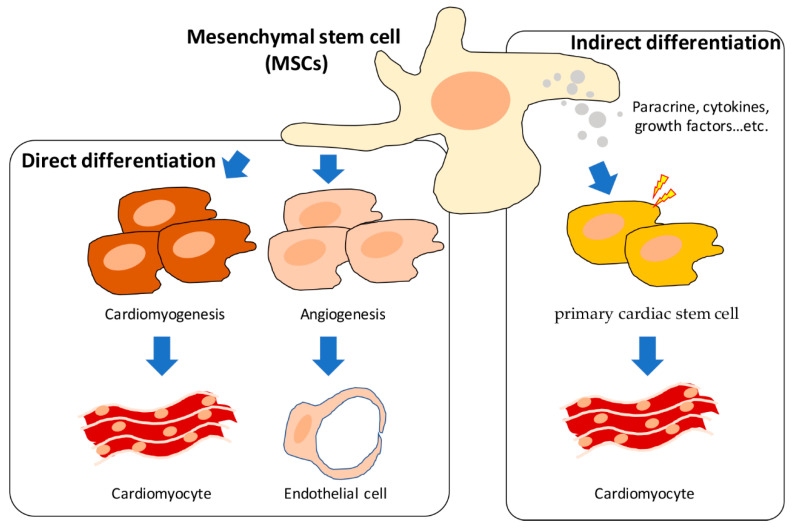
The direct and indirect differentiation of mesenchymal stem cells (MSCs) in cardiac tissue regeneration.

The multi-lineage differentiation of mesenchymal stem cells (MSCs) has been reported, and the related research accumulated, over decades. The application of MSCs to heart-related tissue regeneration and disease treatment has also developed rapidly in the last 10 years. For now, there are two main hypotheses: (1) the direct differentiation of MSCs into cardiomyocytes and endothelial cells through cardiomyogenesis and angiogenesis, and (2) indirect regulation by the primary cardiac cells through paracrine, soluble cytokines, and growth factors (Figure 1) [21,22]. 

Early in 1999, the ability of bone marrow cells (BMCs) to differentiate to cardiomyocytes was proven by Tomita, through the autologous transplantation of bone marrow cells (BMCs) into ventricular scar tissue, which pushed the BMCs to differentiate and restored the damage induced by myocardial function [23]. The cardiomyogenic differentiation of BMCs improved the cardiac function in the scar tissue of adult rats’ ventricles and responded to angiogenesis in both in vitro and in vivo models. The cardiac-like cells differentiated into from BMCs were identified to be cardiomyocytes by Toma in 2002, by evaluating the expression of desmin, beta-myosin heavy chain, alpha-actinin, cardiac troponin T, and phospholamban [24]. The results suggest that BMCs provided a base cell, and could be an alternative strategy for replenish of myocardium to improve cardiac function. The cardiac-differentiation of MSCs has been applied in a swine myocardial infarction (MI) model. By directly injecting the MSCs into the region of the piezoelectric crystals of the ventricle, the implanted MSCs were able to be labelled and measured based on their differentiation in vivo. MSC engraftment and cardiomyocyte-specific protein expression were evaluated by the expression of α-actinin (α-act), troponin T (TnT), phospholamban (PLB), and tropomyosin (TM) in treated myocardium [25]. From these investigations, MSC engraftment for myocardial regeneration is believed to be a promising therapeutic strategy for heart failure patients (Table 1) [26].

An early boost of the involved mechanisms, such as intracardiac regeneration and angiogenetics, can be used to improve the differentiation of MSCs, and consequently the healing outcomes of cardiac diseases. Epicardial erythropoietin (EPO) has been shown to promote MSCs’ cardiomyogenic differentiation and angiogenetic activities through the enhancement of paracrine activity. The early postischemic biomarker of MSCs in MI-triggered cardiac proliferation presents on CD45^−^CD44^+^DDR2^+^ on the surface of the cell membrane and exhibits multi-lineage differentiation abilities, including typical clonogenicity with colony-forming unit-fibroblast formation, and adipogenic, chondrogenic, and osteogenic differentiation [27]. Moreover, an EPO intramyocardial epicardial injection showed evidence of an increased level of intracardiac regenerative key indicators, including stromal cell-derived factor 1 (SDF1), C-X-C Motif Chemokine Receptor 4 (CXCR4), CD34, Bcl-2, cyclin D1, Cell Division Cycle 2 (Cdc2), and Matrix Metallopeptidase 2 (MMP2), as well as activating the signaling pathway of transforming growth factor β (TGF-β)/WNT, AKT, and its upstream regulator FOS and frizzled family receptor 7 (FZD7) [28]. MSCs significantly improved the proliferation of intracardiac mesenchymal after MI for 24 h [28]. Accumulated evidence suggests that transplantation of MSCs not only stimulates resident cardiac stem cells, but also contributes to the rebuilding of blood vessels after MI. However, several issues remain to be solved. Poor cellular nourishment occurs following insufficient blood supply [29]. On the other hand, MSCs have the ability to promote angiogenesis by differentiating into endothelial cells, and in this way can reverse the fibrosis caused by tissue damage [30,31]. However, the exact regulation and interaction of MSCs and primary cardiac cells are also controversial, due to inconsistent therapeutic efficacy.

Mesenchymal stromal cells have also shown potential as an option for cardiovascular disease therapy. It was found by Liao that Nestin positive cardiac mesenchymal stromal cells (Nes^+^ cMSCs) exhibit the ability of self-renew and engage in tri-lineage differentiation [32]. A crucial factor, Periostin, which is highly expressed in Nes^+^ cMSCs, was shown to be positively correlated with the therapeutic efficiency of mesenchymal stromal cells, due to the induction of anti-inflammatory M2 phenotype macrophages and polarization in ischemic myocardial regions. Early in 2007, it was found that the enhancement of Nestin expression could provide benefits in promoting resident cardiac stem cells’ spontaneous regenerative processes after MI occurrence [33]. For now, Nestin has been considered a positive marker in ischemic damage-caused ventricular cardiomyocytes, and its role in supporting cell cycle progression has been demonstrated [34]. Not only in mesenchymal stromal cells, but also in bone-derived mesenchymal stem cells, Nestin has been shown to positively regulate cardiac function in an acute myocardial infarction mouse model. Bone marrow stem cells (BMSCs) enhance the migration of endogenous endothelial cells through activation of chemokines, such as through the CXCL12/CXCR4 chemokine pathway, and through the secretion of TIMP-1/2 in ischemic heart post-MI [35]. These results strongly suggest the role of Nestin as a useful biomarker of stem cells for cardiac tissue regeneration.

A better treatment efficacy for mesenchymal stromal cells than mesenchymal stem cells has been concluded, and the activation of Periostin and the polarization of M2 macrophages appears to be the decisive key elements for cardiac healing after AMI. In 2007, the role of Periostin in heart regeneration was reported. Periostin has been shown to induce the proliferation of cardiomyocytes and improve cardiac function. Further evidence has indicated that the enhancement of proliferation by Periostin requires integrin α_v_ and a β_1_, β_3_, or β_5_ subunit to accelerate the cell cycle progression [36]. Clear evidence shows that the Periostin-peptide triggers the formation of myocardium strips within the infarct scar, leading to the local improvement of myocardial function [37]. Conversely, the deletion of Periostin impairs cardiac regeneration post-MI. The involved molecules, including elevated glycogen synthase kinase (GSK) 3β and decreased phosphatidylinositol 3-kinase (PI3K), have been identified, as well as the downstream regulators phosphorylated serine/threonine protein kinase B (p-Akt) and cyclin D1 [38]. These results suggest the role of Periostin in positively regulating cardiac regeneration after MI occurrence. 

**Figure 2:**
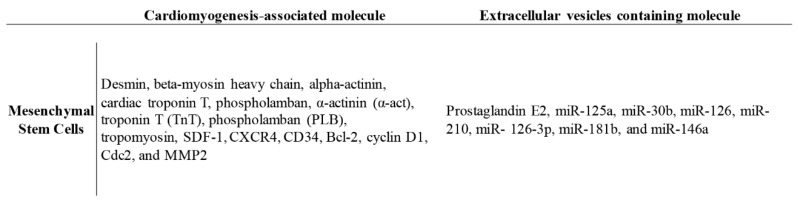
The potential molecules involved in the progression of cardiomyogenesis and/or contained in the extracellular vesicles that regulate MSC functions.

There are more and more reports regarding the paracrine functions provided by MSCs. Among them, secreted soluble factors and extracellular vesicles (EV) contain exosomes and microvesicles. The cargo exists in predominantly endosomal regions, such as miRNA, mRNA, proteins, and even lipoproteins, and are responsive to the therapeutic effect of MSCs (Figure 2) [39,40]. It was found that lipids, including large amounts of ceramide, cholesterol, and sphingolipid, are covered in the microvesicles and delivered to target cells through lipid–ligand receptor interactions [39]. However, most reports suggest that MSCs-EVs provide cardioprotection and promote cardiac regeneration by decreasing oxidative stress and the inflammation response, and increasing neoangiogenesis and pro-survival signaling, as shown in a MI mouse model [40,41,42]. 

The mechanisms of MSCs’ functions in cardiac tissue repair and inflammatory inhibition after injury have not yet been fully clarified, and they are currently used for the treatment of cardiovascular diseases-associated mouse model based on autologous transplanted stem cells, including promoting the cardiomyogenic and vasculogenic effects of cardiac stem cells. More evidence and studies are required to clarify the mechanisms by which MSCs achieve heart repair, through shedding paracrine and microvesicles into resident primary cardiac cells.

## 3. The Regulatory Effect of Lipids on MSCs in Cardiac Regeneration

Lipids have been considered key mediators that can regulate cellular processes, and may induce multiple signaling pathways [43,44]. In addition, the use of lipid metabolites as cell biomarkers can indicate different biological states and cell activities [45,46]. The total content of lipids in the cell is the lipidome. Any modification in the cellular lipidome induces or regulates signaling associated with cell function.

More and more reports have been released regarding the lipid profile of MSCs recently, and the role of lipoproteins in altering MSC functions and properties, including their proliferation capacity, immune-modulatory functions, immunomodulatory regulation, and the differentiation potential of multiple lineages [47,48], although most of these investigations have focused on the effect of lipids in regulating MSCs’ adipogenic differentiation capability. However, uncertainty persists regarding the relevance of MSC differentiation through cardiomyogenesis and vasculargenesis. Lipid metabolites are also correlated with the risk of myocardial infarction and is chemic stroke. To date, lipoproteins, including very low-, intermediate-, and low-density lipoprotein (VLDL, IDL, and LDL) particles, have been reported to be positively associated with cardiovascular diseases such as MI and ischemic stroke (IS) [49]. In contrast, high-density lipoprotein (HDL) particles have been shown to be inversely correlated with MI occurrence. The process of adipocyte formation, also known as adipogenesis, is regulated by a network of complex molecular processes, and this regulatory mechanism may also be affected by lipoproteins [50]. Thus, we assume that lipoproteins regulate differentiation into cardiomyocyte and endothelial cells, thereby affecting heart tissue regeneration. However, the effect of lipids in MSC function regulation has not yet been fully discussed. 

## 4. Phospholipids Regulation of MSC Function

Lipoproteins include cholesterol, triglycerides, and phospholipids. These blood lipids are fat-soluble, and must be combined with plasma proteins to form lipoproteins before they can be transported to various organs and tissues by the blood. A total of 20–30% of the plasma in the blood is transported by high-density lipoproteins, mainly to bring the plasma of the surrounding tissues back for insulin metabolism. The higher a person’s level of HDL, the lower their chance of having coronary heart disease, so HDL-cholesterol is called “good” plasma. 

The main content of HDL is phospholipids, including phosphatidylcholine (PC), phosphatidylethanolamine (PE), phosphatidylserine (PS), and phosphatidylinositol (PI). Sphingolipids and cholesterol primarily serve as building blocks for membranes and organelles [51]. Polyunsaturated fatty acid (PUFA)-containing lipid species increase in the mature stages of mesenchymal stromal cells derived from human fetal membranes (hFM-MSCs). Chatgilialoglu et al. found that culturing hFM-MSCs in vitro changes their fatty acid composition. In order to maintain and improve the functional properties of hFM-MSCs, tailored lipid supplements can mimic membrane environments similar to its physiological counterparts. Stem cells can also be used in patients to achieve therapeutic outcomes [52]. The results also suggest that there is an association between phospholipids and mesenchymal stromal cell differentiation. 

**Figure 3:**
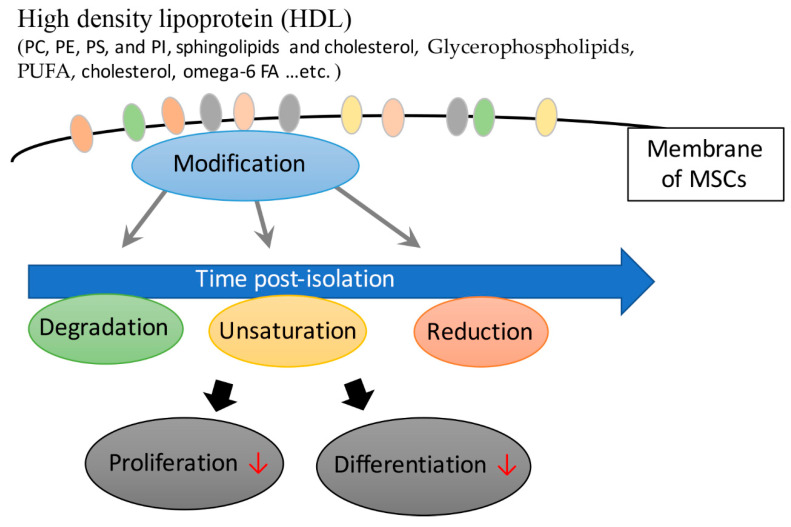
The positive regulatory role of high-density lipoprotein (HDL) in maintaining MSC function and differentiation.

Sphingolipids and phospholipids constitute an independent class of lipids, but they exist in similar membranes, and their overlapping functions are generally similar to those of phospholipids with similar biosynthetic pathways. Phospholipases are their main digestive enzymes, responsible for regulating many important physiological processes (including the production of large amounts of signaling lipids), and they seem to affect all diseases in some way [53]. The glycerophospholipid profiles of human bone MSCs from young and old donors and across passages following in vitro culturing have been specifically assessed [52,54]. The report pointed out that when MSCs were isolated from young donors, the content of total PI and total lysoPC were significantly higher compared with the elderly. Small changes in membrane glycerophospholipids may alter lipid derivative-mediated signaling, with serious consequences. Therefore, when stem cells are used for therapy, adjustment of changes caused by in vitro culture conditions becomes extremely important [54]. Freshly isolated MSCs from bone marrow carry high levels of omega-6 FA, which decrease with the gradual decline of MSC function and differentiation activity [52]. These findings indicate that a tailor-made MSC medium is necessary; this medium could minimize the changes of FA composition in MSCs to maintain their original function and activity (Figure 3) [52]. 

However, Papsdorf also presented evidence that the unsaturation of membrane phospholipids increases with age and increasing peroxidation products may cause more unexpected cell damage. Lipid metabolism is essential for cells to adapt to external conditions, proliferate, and even differentiate. There is also an interaction between lipid metabolism and the cellular chromatin state [55]. The mechanisms by which changes in lipid metabolism could contribute to age-related changes in stem cell behavior remain largely unexplored. 

## 5. Cholesterol Regulation of MSC Differentiation

Sixty to seventy percent of cholesterol in the blood is carried by low-density lipoproteins, mainly to bring cholesterol from the liver to the surrounding tissues. Hypercholesterolemia caused by excessive low-density lipoprotein cholesterol is a risk factor for coronary atherosclerosis and heart disease, so low-density lipoprotein cholesterol is called “bad” cholesterol.

It has been reported by Sohn et al. that supplementing cholesterol to MSCs increases membrane cholesterol and leads to a decrease in membrane fluidity, and an increase in the expression level of caveolae and CAV-1 in the cell membrane. The expression of α1, α4, and β1 integrin will also increase in a dependent manner and have a higher adhesion rate to fibronectin and collagen. Conversely, reducing the expression of CAV-1 in MSCs leads to a decrease in cholesterol levels, and due to the reduced delivery of cholesterol into the cell membranes of MSCs, the membrane fluidity is increased. The expression of CAV-1 and the related signaling proteins in MSC cells are reduced, as well as the adhesion speed to different substrates. These results indicate that the perturbation of cholesterol/CAV-1 levels will significantly affect the membrane properties of MSCs. These findings confirm that the modification of membrane cholesterol and/or CAV-1 and caveolae can be used to manipulate the biological activity of MSCs, which in turn affects their differentiation features [56]. 

**Figure 4:**
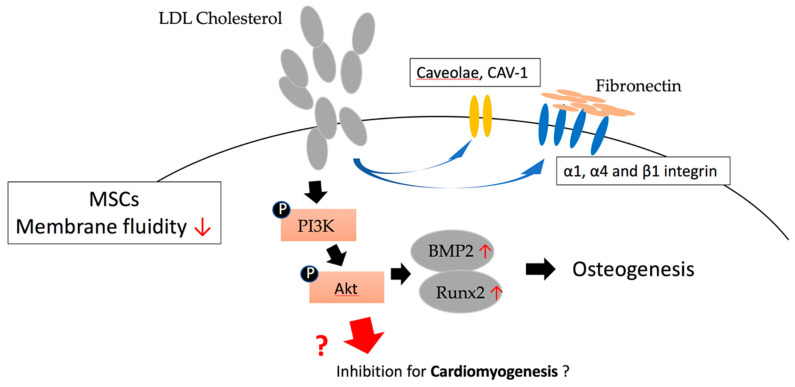
The effect of low-density lipoproteins (LDL) cholesterol in regulating MSC differentiation through the phosphorylation of PI3K/Akt pathway.

The related mechanism of how cholesterol regulates MSCs was reported by Baker et al. They showed evidence that 10 days after the differentiation of MSCs into osteoblasts, phosphorylated Akt began to accumulate in caveolae. Moreover, LY294002 and Akt siRNA inhibited the osteogenesis of the MSCs, confirming the importance of the PI3K/Akt pathway in MSC differentiation. In addition, MβCD inhibited the osteogenesis of MSCs. Conversely, Cav-1 siRNA and cholesterol oxidase can transfer Cav-1 from the caveolae, but instead enhanced Akt-induced osteogenic signaling. In addition to proving that PI3K/Akt signaling is a key pathway required for human MSCs to differentiate into bone, this result also shows that phosphorylated Akt in non-caveolar membrane rafts has a positive effect on MSC differentiation, but a negative impact while in caveolar membrane rafts [57]. It has also been reported that, during the differentiation of MSCs initiated by cholesterol stimulation, the mRNA and protein levels of the osteogenic lineage marker alkaline phosphatase (AKP) are increased, enhancing their activity and mineralizing nodules. In contrast, ACAT (acyl-CoA: cholesterol acyltransferase) inhibitor Sandoz58035 and SiRNA-ACAT1 can inhibit free cholesterol (FC) esterification to cholesteryl ester (CE), thereby inhibiting the cholesterol-induced stimulation response. In addition, BMP2 and Runt-related transcription factor 2 (Runx2) also play important roles in cholesterol-induced MSC osteogenesis. This indicates the possibility of CE in the cholesterol-mediated MSC osteogenesis process, and also reveals the importance of cholesterol in regulating the differentiation of MSCs (Figure 4) [58]. Obniski et al. showed evidence that dietary lipids can control intestinal endocrine cells produced by Drosophila intestinal stem cells. Cholesterol affects the differentiation of stem cells by changing the level of Notch signaling, and through changes to its transport in endosomal vesicles. Lipid-mediated Notch signaling occurs in many other tissues, indicating that Delta trafficking in many cells is sensitive to cellular sterol levels [59]. 

## 6. Triglyceride (TG) and Steroids in MSC Differentiation

**Figure 5:**
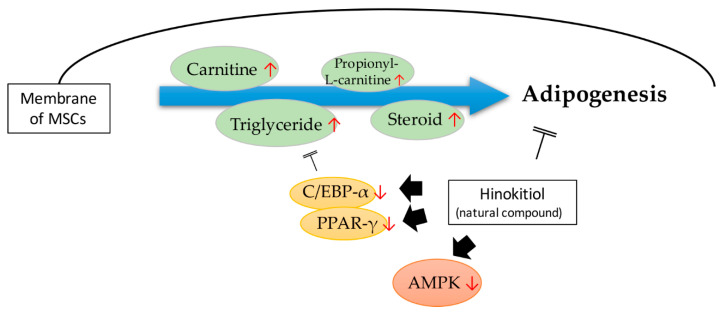
The effect of very low-density lipoprotein (VLDL)-containing TG in inducing MSC adipogenesis, and the type of induction that could be inhibited by the natural compound hinokitiol.

Most of the research conducted so far has shown that triglycerides float upon the different stages of MSC differentiation, such as adipogenesis. Recently, by using in-house produced 13C isotopically labeled compounds, hyphenated high-end mass spectrometry (high-resolution Orbitrap MS), and chromatography (HILIC, RP), a general low abundant amino acid was found in the mesenchymal stem/stromal cells’ transition to progenitors of adipocytes. Further, TG and lipid precursors such as carnitine and propionyl-L-carnitine were found to increase during MSC differentiation [60]. Likewise, a natural compound, hinokitiol, has the capability of reducing lipid accumulation and TG content in MSCs, and impairs MSC adipogenesis. The main mechanism analysis revealed the de-regulation of C/EBP-α and peroxisome proliferator-activated receptor γ (PPAR-γ) expression, as well as the inhibition of AMPK, induced by hinokitiol. Autophagy activator rapamycin can specifically reactivate hinokitiol-induced MSCs adipocyte de-differentiation. AMPK de-phosphorylation can also reduce the suppression of autophagy and adipogenesis induced by hinokitiol (Figure 5) [61]. 

Steroids impact greatly on the differentiation of bone marrow stem cells (BMSCs). Our group first reported steroid-induced adipogenesis of MSCS in a murine bone marrow pluripotential cell line, D1. Steroid-induced adipogenesis by BMSCs in marrow may influence the development of osteonecrosis of the femoral head (ONFH) [62]. Steroid treatment may also affect the fate of BMSCs. D1 cells produce adipocytes when transplanted into steroid-treated mice [63]. Alcohol and glucocorticoids are risk factors associated with ONFH [64]. In addition to steroids, alcohol may suppress Wnt/beta-catenin signaling in human BMSCs, reducing the osteogenic differentiation ability and increasing the adipogenic differentiation ability, with more lipid droplets in cells [65]. The MSCs behaved differently in patients with and without osteonecrosis. 

BMSCs from patients with glucocorticoid-induced osteonecrosis (ON) possessed less osteogenic gene expression and less osteogenic differentiation, whereas BMSCs from patients with alcohol-induced ON possessed more adipogenic gene expression and more adipogenic differentiation. Dysfunction of BMSCs may be one of the causes of ONFH, with differing dysfunctions in alcohol-induced ON and glucocorticoid-induced ON. Glucocorticoids may have more of a suppressive effect on osteogenesis than alcohol, whereas alcohol may have a more potent adipogenic effect than glucocorticoids on BMSCs [66].

More molecules involved in MSC differentiation were discovered in the MSC treatment of the steroid-induced ONFH model. Steroids are known to inhibit osteogenic differentiation and promote adipogenesis, through the significant increase of the gene expression of AP2 and PPARγ, and the decrease of RUNX2 and type I collagen (Col I) in MSCs. More importantly, the TG level has also been found to increase during differentiation modification, indicating the participation of TG in the condition of MSCs treated in steroid-induced ONFH; thus, osteogenesis is implicated and adipogenesis is improved [67]. The results suggest that the TG level might be associated with the differentiation status of MSCs, but the regulatory pathway between TG and MSCs still needs to be further clarified.

## 7. Lipid-Lowering Drug Regulation of MSC Function and Differentiation

Treatment of dyslipidemia can reduce the risk of heart disease by 30% for 5 years. For patients with cardiovascular disease, the benefits of lipid-lowering therapy have been clearly demonstrated. However, dyslipidemia is common among people without CVD who are classified as not requiring treatment. Certain corresponding ethnic groups and genders have an increased risk of getting CVD. Therefore, a lot of research is needed to improve and optimize the management and treatment of dyslipidemia [68]. Herein, we will discuss the modulation and effects of lipid-lowering drugs on MSCs. 

HMG-CoA reductase inhibitor (3-hydroxy-3-methyl-glutaryl-coenzyme A reductase inhibitors, statins) can effectively reduce plasma total cholesterol and LDL-C concentration by inhibiting liver cholesterol synthesis and increasing the number and activity of liver cell LDL-receptors, which can reduce the incidence of coronary heart disease and mortality.

**Figure 6:**
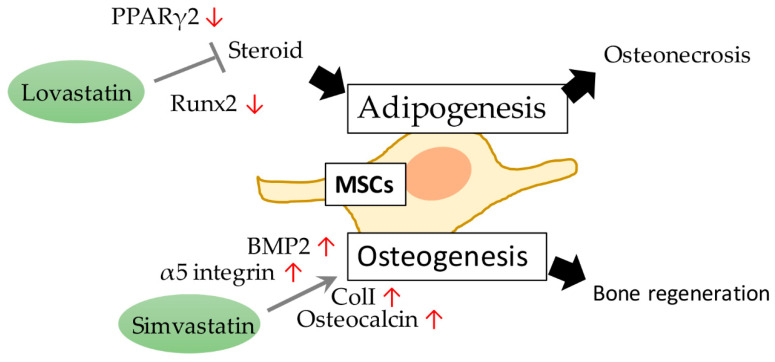
The lipid-lowering drugs lovastatin and simvastatin prohibit adipogenesis and osteogenesis, respectively, to manipulate MSC cellular processes.

Our colleague first found that lovastatin prevents steroid-induced adipogenesis and osteonecrosis in chickens. Lovastatin can inhibit steroid-induced fat specific gene expression, and counteracts the inhibitory effects of steroids on osteoblastic gene expression in BMSCs [69]. Steroid-induced adipogenesis in the marrow may contribute to osteonecrosis, and lovastatin may be helpful in preventing the development of steroid-induced osteonecrosis in chickens [70]. Lovastatin inhibits adipogenic and stimulates osteogenic differentiation by suppressing peroxisome proliferator-activated receptor γ2 (PPARγ2) and increasing Runx2 expression in BMSCs [71]. We discovered in 2009 that taking simvastatin (SVS) helped to restore trabecular bone volume and osteoblast number in an ovariectomized rat model, and bone marrow stem cells (BMSCs) could be involved in this (Figure 6). SVS oral treatment increased the bone mass and the number of osteoblasts in the distal femur, proximal tibia, and vertebrae of ovariectomized (OVX) rats. In addition, after SVS treatment, osteoblasts with immunostained BMP2, type I collagen, and osteocalcin in the vertebrae of OVX rats increased significantly (Figure 6). The results of this study confirm that SVS can enhance the production of osteogenic proteins in bones, and can be used to help prevent bone loss in OVX rats [72]. We also demonstrated that during the osteogenesis induced by SVS, it is essential to maintain a complete actin cytoskeleton and enhance cell rigidity. Simvastatin acts as an osteoinductive factor and promotes bone marrow stem cell-based bone regeneration by increasing actin filament organization and cell rigidity [73]. 

The functional molecule analysis of the effect of SVS on BMSCs was carried out using in vitro mouse BMSCs D1 cells. The results showed that SVS can significantly increase the expression level of osteogenic marker genes in D1 cells, as well as the subsequent ALP activity and calcium deposition, in a dose-dependent manner. In addition, the expression level of α5 integrin protein increased as p-FAK was up-regulated. When we reduced the expression of α5 integrin in D1 cells, significant inhibition of SVS-induced osteogenic genes expression was found, and a reduction of the subsequent ALP activity and calcium deposition. Therefore, these data suggest the key role of SVS in inducing osteogenic differentiation of BMSCs, and also show the feasibility of using SVS to regulate α5 integrin/FAK signaling to manipulate the differentiation of MSCs [74]. We also found that SVS stimulates BMSC osteogenesis through non-GPER-1 mediated ERα signaling, wherein ERα ligands and co-activators enhance ERα-dependent transcriptional activity and thus promote BMSC differentiation [75].

It has been reported by Zanette that SVS could regulate the proliferation and gene expression of mesenchymal stromal cells. SVS negatively regulates mesenchymal stromal cell proliferation and regulates the expression of proliferation-related genes in a dose-dependent manner. However, it has also been observed that simvastatin increases proliferation actively. The decrease in mesenchymal stromal cell size and the increase in pluripotency may be related to the evidence that statins can prevent cell aging senescence [76]. It is speculated that there may be a smaller subpopulation of mesenchymal stromal cells that can be induced by SVS, thereby increasing the activity maintaining the entire mesenchymal stromal cell pool and protecting it from cell senescence in a long-term cellular process. This result helps to explain the pleiotropic effects of statins, and their potential to induce stem cell tissue regeneration [76].

## 8. Lipid-Lowering Drugs Promote the Potential of MSCs in Cardiac Tissue Regeneration

**Figure 7:**
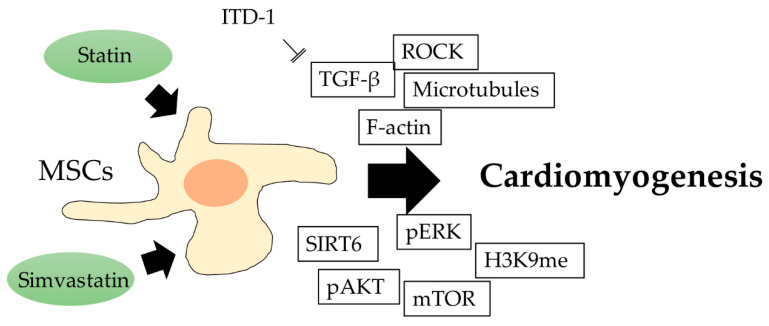
The schematic diagram showing lipid-lowering drug regulation of MSC cardiomyogenesis, and the potential involved molecules.

The molecules involved in the cardiomyogenesis of human MSCs were reported by Ghosh et al. Cardiomyogenic differentiation through F-actin, microtubules, and Rho-associated coiled-coil kinase (ROCK) protein is promoted by aligned fibrous scaffolds, and functional molecules, including activated ERK, AKT, and mTOR, were found to be involved during differentiation. The sirtuin family of NAD-dependent enzymes was also shown to participate in the regulation of MSC cardiomyogenesis, and an increased level of SIRT6 results in the enhancement of differentiation, which was associated with a decreased level of H3K9 [77]. Small molecule ITD-1 is a highly selective TGF-β inhibitor. ITD-1 treatment confirmed that TGF-β is involved in the process of ESC mesoderm formation and cardiac differentiation. ITD-1 selectively enhances the differentiation of uncommitted mesoderm into cardiomyocytes, rather than the differentiation of vascular smooth muscle and endothelial cells. This also shows the importance of TGF-β and its related signaling in controlling the differentiation of pluripotent precursor cells into myocardium (Figure 7) [78].

Yang et al. found evidence of the potential of SVS in promoting heart tissue regeneration. Using a Chinese miniswine AMI model, they found that SVS significantly improved the treatment effect of MSCs in cardiac function and decreased the perfusion defects. Compared with the control group, the cell apoptosis rate in the SVS group and the SVS with MSC transplantation group was significantly lower. In addition, in both the SIMV group and the SVS + MSCs group, oxidative stress and inflammation were significantly reduced in the infarct area. These results also show that SVS treatment can improve the efficacy of MSC transplantation in acutely infarcted hearts by promoting cell survival and cardiovascular differentiation [79]. Moreover, SVS has been reported to regulate the protein level of PAI-1 and the cascade signaling pathway. SVS inhibits PAI-1 expression, and exhibits therapeutic potential against injury by modulating the main processes of an inhospitable environment in regards to fibrosis and cell migration [80]. Simvastatin’s process of regulating cell function plays an important role in the homing and implantation of cells after MSC cell therapy. Based on this regulatory effect, SVS can be used as an adjuvant treatment or enhancer in different cell therapies. The modification in the tissue microenvironment also promotes the efficiency of MSC homing due to the enhanced paracrine effect of stem cells during tissue regeneration [80].

In addition, Zhang et al. confirmed through a hind limb ischemia experiment in mice that the statin group significantly improved the ratio of hind limb blood reperfusion compared with the control group and had significantly higher capillary density. The combined use of statins and MSCs can further improve revascularization and led to the highest capillary density in all groups. The use of GFP-labeled transplanted cells further confirmed that in the statin-MSC group, low-dose simvastatin cooperated with MSC to enhance functional neovascularization in a hind limb ischemia mice model. This is strong evidence for the role of statins in regulating MSC angiogenesis [81].

However, there is also evidence against the use of SVS in MSC-initiated treatment. It was reported by Li that SVS exhibits cytotoxicity to MSCs by reduction of cell viability, accompanied with the down-regulation of the NF-κB p65 protein level. Reverse treatment with an inhibitor of nuclear factor kappa-B kinase subunit beta (Iκκ-β) can prevent SVS-induced cell death. These results imply that if the effect on differentiation is not considered, SVS is toxic to MSCs, and the inhibition of NF-κB translocation could be important [50].

## 9. Conclusions

Considering the evidence gathered to date, the use of MSCs in regeneration of the cardiomyocyte and vascular endothelial cells of cardiac tissue is encouraging. The more we understand the relevant mechanisms involved, the more likely we are to find new ways to treat myocardial infarctions or any other CVDs, so that critical patients have other options before requiring a complex and high-risk heart transplantation. However, the content of blood lipids/lipoproteins may affect the success rate of MSC-based tissue regeneration. Studies have shown that we should not ignore the influence of lipids/lipoproteins on MSC cellular processes. Therefore, actively understanding the role of lipid/lipoprotein in MSCs will greatly benefit and improve regenerative medicine, especially relating to those tissues largely utilizing lipid as energy source. The positive regulation of statins in MSCs is impressive, and the results suggest the potential of lipid-lowering drugs in promoting stem cell-mediated regenerative medicine of cardiac tissue.
